# Human Immunodeficiency Virus (HIV)/Hepatitis B Virus (HBV) Coinfection and Diffuse Interstitial Lymphocytosis Presenting With Kidney Disease

**DOI:** 10.7759/cureus.71765

**Published:** 2024-10-18

**Authors:** Sandeep Sasidharan, Eugene K Yeboah, Surya V Seshan, Thin Thin Soe, Subodh J Saggi

**Affiliations:** 1 Nephrology, State University of New York Downstate Health Sciences University, Brooklyn, USA; 2 Internal Medicine, State University of New York Downstate Health Sciences University, Brooklyn, USA; 3 Pathology and Laboratory Medicine, Weill Cornell Medicine, New York, USA

**Keywords:** combined nephropathy in hiv-hbv coinfection, diffuse interstitial lymphocytosis (dil), hbv-associated nephropathy (hbvan), hepatitis b-associated membranoproliferative glomerulonephritis (mpgn, hiv associated nephropathy (hivan)

## Abstract

This case report highlights a rare presentation of acute kidney injury (AKI) with nephrotic syndrome in a Pacific Islander with concomitant acute HIV and hepatitis B virus (HBV) coinfection, progressing to require hemodialysis; the patient recovered completely and discontinued dialysis after a course of high-dose steroids and initiation of antiviral agents. The renal biopsy revealed features consistent with HIV-associated nephropathy (HIVAN) and HBV-associated nephropathy (HBVAN), along with diffuse interstitial lymphocytosis (DIL) showing dominant CD8 lymphocyte infiltration and high Hep B and HIV viral loads. Management challenges included the decision on the initiation of antiviral agents simultaneously with immunosuppressive agents. DIL syndrome (DILS) has become exceedingly rare since the advent of highly active antiretroviral therapy (HAART). To our knowledge, this is the first reported case of combined nephropathy in HIV-HBV coinfection.

## Introduction

Human immunodeficiency virus (HIV) and hepatitis B virus (HBV) are two common chronic viral infections in the United States (US). In 2021, 36,136 people received an HIV diagnosis, and 2,045 acute HBV cases were reported, amounting to an estimated 13,300 infections. The prevalence of both chronic infections has remained steady over the decade, with 1.2 million cases of HIV and 2.4 million cases of chronic HBV infections. Globally, an estimated 8-10% of people with HIV have chronic HBV infection, although the prevalence of coinfection varies significantly by region [1,2]. An estimated 5-10% of people with HIV in the US have chronic coinfection with HBV [2].

Hepatitis B-associated membranoproliferative glomerulonephritis (MPGN) is an immune complex GN, characterized by nephritic sediment, hypocomplementemia, and moderate proteinuria with acute kidney injury (AKI) [1]. A rare manifestation, the diffuse infiltrative lymphocytosis syndrome (DILS), characterized by CD8(+) T-cell lymphocytosis and infiltration of multiple organs, has been described in HIV-infected individuals and is often associated with uncontrolled or untreated HIV infection [2,3]. However, the incidence of DILS has also become increasingly rare with the advent of highly active antiretroviral therapy (HAART). We report the first documented case of concomitant HIV and Hep B coinfection associated with membranous nephropathy and DILS involving the kidneys in a 64-year-old male.

## Case presentation

A 64-year-old male immigrant from the Pacific Islands with a past medical history of hyperlipidemia, arthritis, gout, and herniated disc presented to the Emergency Department with one-day abdominal pain, suprapubic tenderness, bilateral lower extremity, right arm, and scrotal swelling. The patient reported intermittent nausea, loss of appetite, and bilious vomiting that had persisted for the past one to two weeks. He had presented to a primary care physician a week before admission due to right arm, scrotum, and bilateral lower extremity swelling and had then been referred to a nephrologist for AKI with proteinuria.

The patient had no known drug allergies and was not taking any medications or supplements. His social history indicated that the patient had immigrated to the USA five years ago. He denied any recent travel or contact with any sick individuals. He also denied alcohol consumption, smoking, and intravenous drug use, but reported having unprotected sexual intercourse with a few women recently. The patient stated that he had been tested several years ago for HIV, and the results were negative. Family history was unremarkable except for type II diabetes mellitus in the patient’s mother.

On physical exam, the patient was alert, and oriented to place and time. He was afebrile with a temperature of 99 °F, tachycardic with a heart rate of 132 breaths per minute, tachypneic with a respiratory rate of 19/minute, saturating well on room air SpO_2_ 100% on RA; he had a BP of 134/90 mmHg, BMI of 28 kg/m^2^, weighed 79 Kg, and he was 1.70 m in height. The abdomen was distended and mildly tender to palpation over the epigastric region. The genitourinary exam demonstrated swelling over the testicles and penis with suprapubic tenderness elicited upon palpation. The lower extremity demonstrated 3+ pitting edema bilaterally reaching up to the mid-thigh.

Investigations

A series of investigations were performed, the findings of which are presented in Table 1.

**Table 1:** Laboratory investigations

Parameter	Patient value	Reference range
Comprehensive metabolic panel		
Sodium	135 mmol/L	136-145 mmol/L
Potassium	5.7 mmol/L	3.5-5.1 mmol/L
Calcium	6.5 mg/dL	8.2-10.0 mg/dL
Chloride	110 mmol/L	98-107 mmol/L
Magnesium	2.0 mg/dL	1.9-2.7 mg/dL
Phosphorus	6.7 mg/dL	2.5-5.0 mg/dL
Creatinine	5.1 mg/dL	0.7-1.3 mg/dL
Blood urea nitrogen	56 mg/dL	7-25 mg/dL
CO_2_	16 mmol/L	21-31 mmol/L
Glucose	78 mg/dL	70-99 mg/dL
Anion Gap	15 mmol/L	10-20 mmol/L
Estimated glomerular filtration rate	22.1 ml/min/1.73m²	>60 ml/min/1.73m²
Liver function test		
Total bilirubin	0.2 mg/dL	0.3-1.0 mg/dL
Albumin	<1.5 g/dL	3.5-5.7 g/dL
Total protein	6.8 g/dL	6.0-8.3 g/dL
Aspartate aminotransferase	24 U/L	13-39 U/L
Alanine aminotransferase	14 U/L	7-52 U/L
Alkaline phosphatase	52 U/L	34-104 U/L
Lipase	22 U/L	11-82 U/L
Lactate dehydrogenase	243 U/L	140-271 U/L
Complete blood count		
Hemoglobin	5.8 g/dL	14.0-18.0 g/dL
White blood count	13.3 k/μL	3.5-10.8 k/μL
Platelet	507,000 /μL	130-400k/μL
Hematocrit	16.4%	42.0-52.0%
Venous blood gas		
pH	7.29	7.31-7.41
pO_2_	41.5 mmHg	30.0-52.0 mmHg
pCO_2_	39.5 mmHg	40.0-52.0 mmHg
HCO_3_	18.2 mmol/L	23.0-28.0 mmol/L
Urinalysis		
Urine osmolality	314 mOsm/kg	500-800 mOsm/kg
Blood (urine)	Moderate	Negative
Urine creatinine	131 mg/dL	20-320 mg/dL
Protein (urine)	>500 mg/dL	Negative
Urine sodium	18 mmol/L	30-90 mmol/L
White blood cells (urine)	6/hpf	<5/hpf
Urine potassium (random)	46 mEq/L	20 mEq/L
Hyaline cast	33/Ipf	<1/Ipf
Urine chloride	23 mmol/L	30-90 mmol/L
Protein spot	612 mg/dL	<500 mg/dL
Coagulation		
Prothrombin time	11.2 sec	10.8-13.7 sec
Activated partial thromboplastin time	34.6 sec	25-35 sec
International normalized ratio	0.9	<1
Lipid panel		
Triglycerides	256 mg/dL	0-150 mg/dL
Total cholesterol	174 mg/dL	0-200 mg/dL
Low-density lipoprotein cholesterol	113 mg/dL	<99 mg/dL
High-density lipoprotein cholesterol	10 mg/dL	30-85 mg/dL
Anemia workup		
Iron	23 μg/dL	50-212 μg/dL
Total iron-binding capacity	<35 μg/dL	240-450 μg/dL
Unsaturated iron-binding capacity	<55 μg/dL	155-355 μg/dL
Ferritin	528.5 ng/mL	16.0-294.0 ng/mL
Erythropoietin	6.4 MIU/mL	2.6-18.5 MIU/mL
Mineral bone disease		
Vitamin D	<8 pg/mL	18-72 pg/mL
Parathyroid hormone	289 pg/mL	15.0-65.0 pg/mL
Glomerulopathy workup		
Cryoglobulin	Negative	Negative
C3 complement	64 mg/dL	87-200 mg/dL
C4 complement	36 mg/dL	19-52 mg/dL
Serum protein electrophoresis	Chronic inflammatory	No M spike
Urine protein electrophoresis	No M spike	No M spike
Hemoglobin A1C	5.4%	<5.7%
Infectious workUp		
COVID-19	Negative	Negative
Flu	Negative	Negative
HIV 1 Ag/Ab	Positive	Negative
CD4 count	141 cells/μL	500-1200 cells/μL
HIV viral load	>280,000 RNA copies/mL	Negative
Hepatitis A	Negative	Non-reactive
HBsAg	Positive	Non-reactive
HBc IgM	Negative	Non-reactive
HBeAg	Positive	Non-reactive
HB Ab	Negative	Negative
Hepatitis B viral load	>7,700,000	Negative RNA copies/mL
Syphilis RPR	Negative	Non-reactive
Toxoplasmosis IgG	Negative	Negative
Cytomegalovirus	Negative	Negative
Hepatitis C IgG	Negative	Negative
Tuberculosis	Negative	Negative
Cryptococcal antigen	Negative	<1:2

Imaging

**Figure 1:**
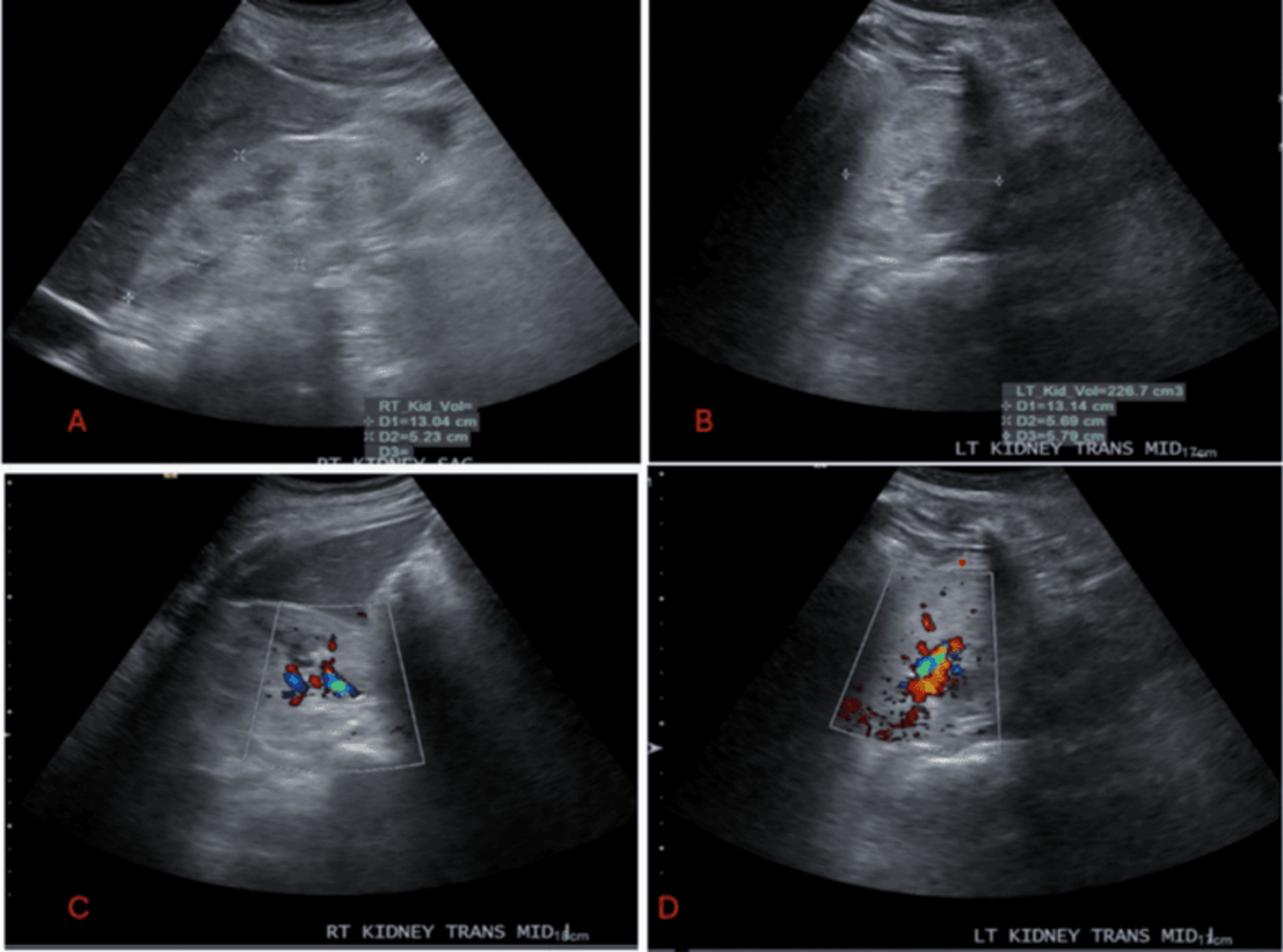
Kidney ultrasound images A. Right kidney with size 13.0 x 5.2 x 6.7 cm, normal parenchyma thickness and contour but Increased echogenicity. No Pelvicalyceal dilatation, calculi, cysts, or solid masses B. Left kidney with size 13.1 x 5.7 x 5.8 cm, normal parenchyma thickness and contour but increased echogenicity. No pelvicalyceal dilatation, calculi, cysts, or solid masses C. Doppler ultrasound of the right Kidney showing normal perfusion. D. Doppler ultrasound of the left Kidney showing normal perfusion

The kidney ultrasound (Figure 1) demonstrated an increase in bilateral echogenicity of the kidneys. The right kidney was measured at 13 cm, and the left at 13.1 cm. No evidence of renal calculi, masses, or cysts was observed. The bladder ultrasound did not demonstrate any focal wall thickening.

Kidney biopsy findings

The kidney tissue samples contained up to 27 glomeruli, none of which were globally sclerosed. Among these glomeruli, seven showed segmental to global wrinkling and collapsing features with focal hyperplastic and vacuolated epithelial cells in the Bowman space. All the glomeruli demonstrated diffuse moderate mesangial proliferative change with increased cellularity and some matrix, encroaching on the capillary lumina. The peripheral capillary walls appeared somewhat irregularly thickened with focal segmental double contours. There was no evidence of endocapillary hypercellularity, necrosis, or crescent formation. There was diffuse active and subacute interstitial inflammation of moderate to severe degree involving the entire cortical tissue examined, composed of lymphocytes, abundant plasma cells (approximately 30%), and macrophages admixed focally with eosinophils with active tubulitis. Nearly 30% of the tubules demonstrated microcystic dilatation containing proteinaceous casts within the lumina. Clusters of proximal tubules also contained increased protein resorption droplets. The rest of the tubules showed shrinkage and simplification suggesting tubular epithelial injury, as evidenced by flattening and extensive loss of brush border with focal sloughing into the lumina. The small arteries and arterioles exhibited mild medial hypertrophy.

Immunofluorescence microscopy (four glomeruli) revealed diffuse, granular 3+ IgG, 1+ IgA, 3+ IgM, 3+ C3, 2+ C1q, and 3+ kappa and lambda light chains along the glomerular capillary walls and mesangial areas in a global distribution. The tubulointerstitial compartment and arterial vessels were negative for immunoglobulins, complement components, and light chains, except for granular 2+ C3 within the vascular walls. Immunoperoxidase stain on paraffin-embedded tissue using monoclonal antibodies to CD3, CD4, CD8, CD20, and CD138 was performed to immunophenotype the infiltrating inflammatory cells. While the majority of the infiltrating inflammatory cells were composed of CD3-positive T lymphocytes, more than 2/3 of them appeared to be composed of CD8-positive cells and the others CD4-positive T-cells. About 5% of the cells appear to be positive for CD20-positive B cells and over 30% of the infiltrating cells were reactive to CD138, indicative of plasma cells.

On electron microscopy, the glomerular capillary basement membranes were markedly and irregularly thickened by numerous coarsely to finely granular subepithelial electron-dense deposits of varying sizes penetrating to the lamina densa along with frequent subendothelial deposits and segmental cellular interposition. The foot processes were totally effaced with numerous microvilli in the urinary space. The overlying visceral epithelial cells were variably swollen containing active cytoplasmic organelles, vacuolization, and protein resorption droplets. The endothelial cells showed the total loss of fenestrations with mild-to-moderate swelling and margination by mononuclear inflammatory cells. Tubuloreticular inclusions are not readily identified within the endothelial cells. The mesangial areas were markedly expanded mainly by cells at the expense of the matrix along with abundant finely granular electron-dense deposits.

**Figure 2:**
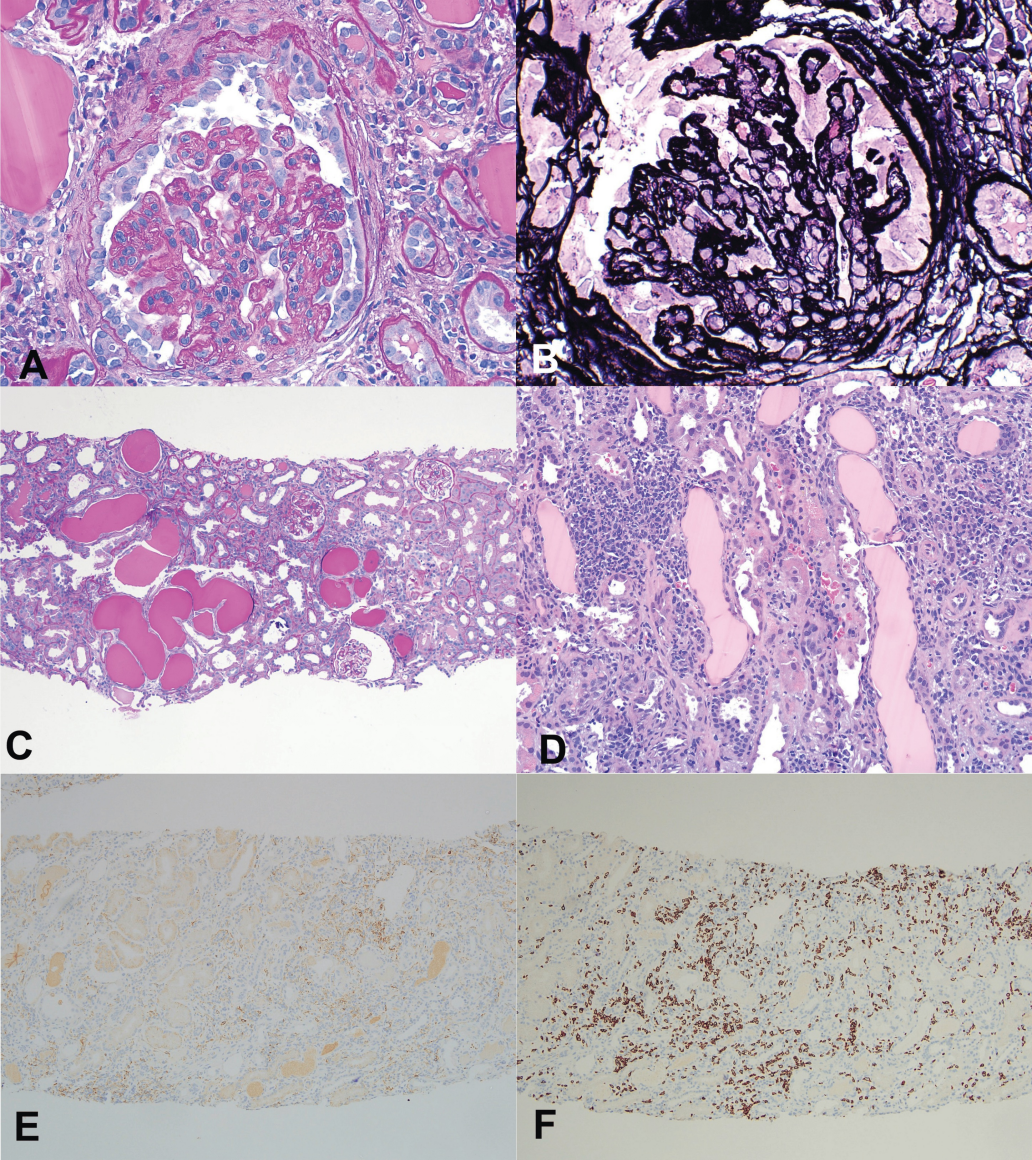
HIV-associated nephropathy A. Collapsing glomerulopathy - glomerulus showing global, marked capillary wrinkling and collapsing features covered by hyperplastic epithelial cells (PAS stain x400) B. Collapsing glomerulopathy - the silver stain of the glomerulus from (A) showing global capillary collapse with occlusion of capillary lumina and hyperplastic epithelial cells (PASM stain x400) C. Diffuse tubulointerstitial disease showing extensive microcystic tubular changes, tubular injury, and interstitial inflammation (PAS stain x100) D. Diffuse lymphocytic interstitial infiltration - moderate to severe interstitial inflammation composed of lymphocytes, plasma cells, and macrophages with adjacent microcystic tubular changes (H&E stain x200) E. Diffuse interstitial lymphocytic infiltration - moderate to severe interstitial inflammation showing positive staining for the CD4 subset of T lymphocytes in a small proportion (immunoperoxidase stain x200) F. Diffuse interstitial lymphocytic infiltration - same area as (C) showing the majority of the cells positive for the CD8 subset of T lymphocytes (immunoperoxidase stain x200)

**Figure 3:**
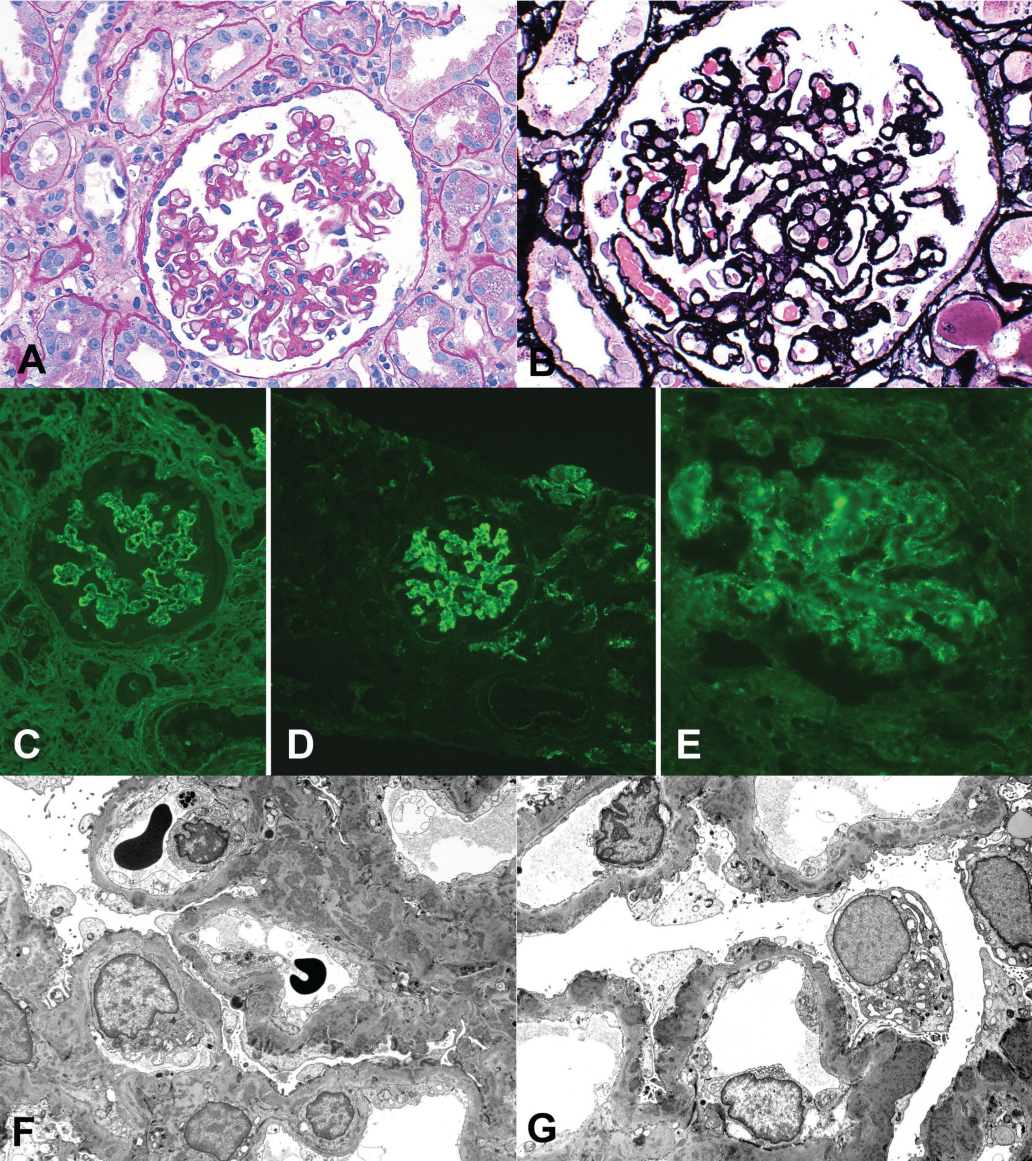
HBV-associated immune complex glomerulonephritis A. Membranoproliferative glomerulonephritis (MPGN) - glomerulus showing irregular thickening of the peripheral capillary walls with focal double contours with mild mesangial cellularity (PAS x400) B. MPGN - the silver stain of the glomerulus from (A) showing irregular thickening of the capillary walls with focal spikes on the outer aspect and double contours in other capillary loops (PASM x400) C. MPGN - immunofluorescence stain of glomerulus showing global, granular 3+ staining for polyclonal IgG along the capillary walls and mesangial areas (FITC antihuman Ig x200) D. MPGN - immunofluorescence stain of glomerulus showing global, granular 3+ staining for C3 along the capillary walls and mesangial areas (FITC antihuman C3 x200) E. MPGN - immunofluorescence stain of glomerulus showing global, granular 3+ staining for C1q along the capillary walls and mesangial areas (FITC antihuman C1q x200) F. MPGN - electron microscopic image of glomerulus showing irregular distribution of focal subepithelial, intramembranous, and subendothelial electron-dense deposits along with abundant mesangial deposits of granular texture (uranyl acetate and lead citrate x5000) G. MPGN - electron microscopic image of glomerulus abundant subepithelial deposits of varying sizes with basement membrane spikes, focal intramembranous, and subendothelial deposits (uranyl acetate and lead citrate x5000)

The kidney biopsy findings are illustrated in Figures 2-3.

Treatment

The patient was given packed RBCs before the biopsy and started on diuresis with Lasix and metolazone after the biopsy. Treatment involving steroids was briefly considered but withheld since the HBV viral load was 7.7 million. The patient was started on Biktarvy (bictegravir/emtricitabine/tenofovir alafenamide), an oral daily tablet to cover HIV and HBV. He had a tunneled dialysis catheter placed and was started on regular intermittent hemodialysis.

Outcome and follow-up

Two weeks after discharge, the patient presented with fever and altered mentation. The workup revealed increased cerebral pressure due to vasogenic edema with lesions in the frontal and partial lobes on the CT head. He was diagnosed with CNS toxoplasmosis. A multidisciplinary team was set up and it was decided to start the patient on high-dose corticosteroids.

The patient was discharged on CNS toxoplasmosis prophylaxis with pyrimethamine, sulfadiazine, and leucovorin for six weeks. After a repeat MRI showed improvement, he was switched to Bactrim for post-toxoplasmosis prophylaxis. The patient had a full recovery after therapy with the steroids and is currently off hemodialysis. Table 2 shows the trend of recovery after initiating treatment.

**Table 2:** Trend of HIV viral load, hepatitis B viral load, creatinine, and eGFR after starting antivirals and steroids eGFR: estimated glomerular filtration rate

HIV viral load trend, UL/ml (month/year)	Hepatitis B viral load trend, UL/ml(month/year)	Creatinine trend, mg/dl (month/year)	GFR trend, ml/min/1.73 m^2^ (month/year)
191000 (initial 03/2023)	7740000 (03/2023)	5.1 (initial: 03/2023)	14 (initial: 03/2023)
282000 (03/2023)	256000 (04/2024)	4.9 (03/2023)	14 (3/2023)
196 (04/2023)	240 (08/2023)	4.6 (03/2023)	16 (003/2023)
403 (05/2023)	<10 (03/2024)	4.7 (03/2023)	15 (03/2023)
117 (06/2023)	<10 (09/2024)	4.5 (03/2023)	20 (03/2023)
120 (08/2023)		4.7 (02/2023)	34 (04/2023)
53 (10/2023)		3.8 (03/2023)	51 (04/2023)
316 (03/2024)		1.3 (04/2023)	48 (04/2023)
<20 (04/2024)		1.4 (05/2023)	65 (05/2023)
46 (most recent 06/2024)		1.2 (most recent: 08/2024)	77 (most recent: 08/2024)

## Discussion

This case report describes a unique presentation of HIV and HBV coinfection associated with progressive kidney disease requiring dialysis. With the advent of HAART, HIV-infected individuals experience prolonged survival, shifting the epidemiological landscape, with a rise in non-AIDS-related complications. Kidney involvement is a significant concern, contributing substantially to morbidity and mortality in this population. The presence of coexisting HBV infection further complicates the clinical picture. HBV can be associated with several glomerular lesions mediated by immune complexes, direct viral damage to kidney cells (cytopathic effects), and in rare cases, medium vessel vasculitis and potential kidney damage from medications used to treat HBV [4-6].

Our patient's renal biopsy revealed features suggestive of both HIV-associated nephropathy (HIVAN) and MPGN, besides the presence of significant interstitial plasma cells, most probably attributed to concomitant other viral infections, such as hepatitis B. Also, the presence of a prominent CD8+ T-lymphocyte infiltrate pointed towards DILS, a rare complication of HIV, generally in those without HAART. DILS is a rare multisystemic syndrome in HIV patients. It is commonly seen in patients of African descent and is often linked to a lack of treatment or poor compliance with HAART medications. APOL1 has a strong association with developing HIVAN in this population [7]. It is characterized by CD8(+) T-cell lymphocytosis associated with a CD8(+) T-cell infiltration of multiple organs. DILS is usually seen in uncontrolled or untreated HIV infection but can also manifest itself independently of CD4(+) T-cell counts. It may present as a Sjögren-like disease that generally associates sicca signs with bilateral parotiditis, generalized lymphadenopathy, and extra-glandular organ involvement. The latter may affect the lungs, nervous system, liver, kidneys, and digestive tract. Sicca syndrome, organomegaly, and/or organ dysfunction associated with polyclonal CD8+ T-cell organ infiltration are greatly suggestive of DILS in people living with HIV [3]. DILS limited to kidney causing dysfunction was seldom encountered when it was prevalent, and, in the current scenario of HAART, it is even more unique.

HIV treatment guidelines recommend immediate initiation of ART in all HIV-positive individuals. Immunovirological control is an important strategy to reduce the incidence of AKI and HIV-related kidney diseases. For proteinuria, ACE/ARB or SGLT2i are recommended [7]. However, there are no randomized trials for them, and these have been extrapolated from studies in other glomerular diseases [8]. The treatment of HBV-associated kidney diseases consists of antiviral therapy in most patients. This recommendation is consistent with treatment guidelines proposed by Kidney Disease: Improving Global Outcomes (KDIGO) [9]. However, there is controversy regarding the use of immunosuppressive therapy in both sets of patients to alleviate kidney disease.

In HBV-associated nephropathy (HBVAN), immunosuppressive therapy, with or without plasmapheresis, is used only in select patients [e.g., rapidly progressive glomerulonephritis (RPGN) or polyarteritis nodosa (PAN) with severe manifestations]. It is recommended that it always be used in combination with antiviral therapy [10-12]. There is scarce data on the treatment of HBV-associated kidney diseases, and these are limited to small case series and clinical trials [10]. In patients with HIVAN, the discussion is more robust, with evidence for the use of steroids emerging from earlier small observational studies that showed improvement in kidney function, reduction in tubulointerstitial inflammation and proteinuria, and slowing progression to end-stage kidney disease (ESKD) [11-14]. However, others caution against the results as these studies were done before or after the early introduction of HAART and were not supported by any recent or large randomized controlled trials.

Thus, in both HIVAN and HBVAN, routine use of steroids is not recommended [8]. Also, there is a risk of adverse events [13]. However, in patients whose kidney function does not improve with therapy, the use of glucocorticoids may be considered on a case-by-case basis, weighing the risks of exacerbation of infection and metabolic derangements against the risk of kidney disease progression. In DILS, a highly inflammatory state, the recommended treatment in this setting is steroid administration, as it is responsive to steroids as well as the institution of regular HAART [2,15-16]. Management of concomitant HIV and HBV infection with DILS presents unique challenges, particularly concerning immunosuppressive therapy. In our patient, we reviewed all the above evidence, and steroid administration was considered for the treatment of DILS but was withheld due to the patient's elevated HBV viral load. The decision reflects the delicate balance required when managing patients with overlapping infectious and immune-mediated renal diseases along with complications of immunosuppression or antiviral medications.

Interestingly, the patient's course demonstrated a potential benefit from corticosteroids administered for CNS toxoplasmosis. While not definitive, this serendipitous observation warrants further investigation into the potential role of judiciously timed steroid therapy in carefully selected cases of coinfection-associated nephropathy with HBVAN and HIVAN. The benefit may also have been due to the resolution of DILS and antiviral therapy reducing the viral loads.

Future research should explore the development of safe and effective treatment strategies for coinfection-associated nephropathy. This may involve tailoring immunosuppressive regimens based on the specific contributions of HIV and HBV to the underlying kidney pathology. Additionally, prospective studies need to evaluate the potential role of antiviral therapy intensification for HBV control, alongside limited-course steroids for HIVAN. Multidisciplinary collaboration among infectious disease specialists, nephrologists, and hepatologists is crucial for optimizing patient outcomes and mitigating the risk of end-stage renal disease.

Limitations

This report involves only a single case, and hence its findings cannot be generalized. Moreover, the retrospective nature of our study limits the ability to establish definitive cause-and-effect relationships between interventions and outcomes.

To sum up, this case report presents a rare and complex presentation of HIV and hepatitis B viral coinfection-associated nephropathy with DILS. It underscores the diagnostic challenges and highlights the need for a multifaceted treatment approach, considering the interplay between HIV and HBV. This report contributes to the limited body of literature on coinfection-associated nephropathy with DILS. The potential benefit observed with corticosteroids in this unique scenario warrants further exploration with prospective studies on this understudied but potentially devastating complication.

## Conclusions

HIV and HBV coinfection is known to affect 5-10% of HIV-positive patients in the US. This is the first known case of HIVAN and HBVAN nephropathy coinfection. However, the exact incidence of this is currently unknown. DILS limited to the kidney is currently a rare presentation thanks to the widespread use of HAART, but clinicians need to be aware of this condition. Management of coinfection nephropathy is complex, and immunosuppression remains a topic of controversy. In our patient, the coincidental use of steroids for CNS toxoplasmosis led to an improvement in kidney function. This potential benefit of steroids needs to be explored further with randomized controlled trials.
